# Normal force of lithium-based magnetorheological grease under quasi-static shear with large deformation

**DOI:** 10.1039/c9ra04987h

**Published:** 2019-08-29

**Authors:** Huixing Wang, Guang Zhang, Jiong Wang

**Affiliations:** School of Mechanical Engineering, Nanjing University of Science and Technology Nanjing 210094 People's Republic of China wjiongz@njust.edu.cn; School of Civil and Environmental Engineering, University of Technology Sydney Ultimo 2007 Australia

## Abstract

An experimental study was conducted to explore the normal force of lithium-based magnetorheological (MR) grease under quasi-static shear. Firstly, MR grease with various carbonyl iron (CI) particles content was prepared. Then the influence of magnetic field strength, CI particles content, shear rate and temperature on the normal force–strain curves for MR grease under a quasi-static monotonic shear condition were investigated. The results exhibit that the normal force of MR grease under quasi-static monotonic shear decreases first and then maintains constant along with the increase of shear strain, and the critical strain is affected by the magnetic field strength and CI particles content. Finally, the normal force *versus* shear strain curves under different magnetic fields and shear strains were obtained by using quasi-static cyclic shear. It was found that the normal force of MRG-70 under quasi-static monotonic shear exhibits peak phenomenon at the beginning of the unloading stage, and the peak value increases with the enlargement of shear strain. Furthermore, when the strain amplitude is higher than 40%, normal force exhibits an abrupt change during the transition from the loading stage to the unloading stage.

## Abstract 2

An experimental study was conducted to explore the normal force of lithium-based magnetorheological (MR) grease under quasi-static shear.

## Graphical abstract

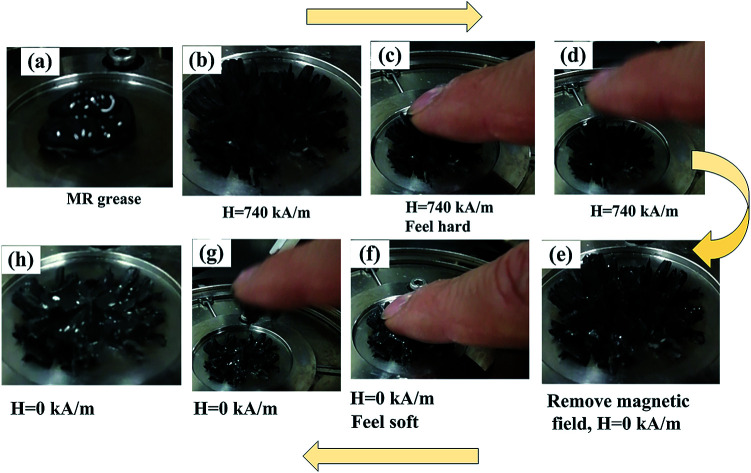

## 1. Introduction

MR grease is a member of the family of MR intelligent materials with tunable rheological properties under the influence of an external magnetic field. Due to the semi-solid state of the grease matrix, MR grease has good sedimentation stability in the absence of magnetic field strength. Meanwhile, when an external magnetic field is applied, the CI particles in MR grease can form a chain or cluster structure, causing a change in the yield stress of MR grease. The above rheological properties of MR grease endow it with outstanding prospects in the application of MR damper,^1–4^ clutch^5–7^ and actuator.^8–10^ Until now, in order to promote the practical application of MR grease, researchers have carried out a series of studies on the rheological properties of MR grease, *i.e.* viscosity, yield stress, storage modulus and loss modulus.^11–16^

The normal force of the MR materials is the force induced by the forming of chain or cluster structure along the direction of the magnetic field which is perpendicular to the shear stress. This normal force has significant impacts on the mechanical performance of MR materials-based devices and is suggested to be considered in the design stage of MR devices.^17^ Vicente *et al.* studied the relationship between normal force and yield behavior of MR fluid by using steady and oscillatory shear test, and they found that the maximum normal force of MR fluid appeared at the position of the suspension began to flow.^18^ See *et al.* conducted an experiment to study the normal force of MR fluid under flow conditions, the results showed that the normal force of MR fluid is correlated with the shear rate. The higher shear rate leads to the smaller shear stress.^19^ Jiang *et al.* tested the influence of shear rate and magnetic field on normal stress of MR fluid under steady shear. The normal stress showed an increasing trend with the increase of magnetic field and shear rate but decrease suddenly at the beginning of shear thickening in MR fluid.^20^ Pang *et al.* investigated the normal stress of MR polymer under large amplitude oscillatory shear, and revealed that the change in normal stress of MR polymer is drastically affected by factors: the Poynting effect and the particle structure.^21^ Ju *et al.* studied the normal force of MR gel under steady and dynamic shear test. It demonstrated that the normal force of MR gel is heavily affected by the external magnetic field. However, with the presence of magnetic field, the dependence of normal force on time, shear rate and frequency are not obvious.^22^ The normal force of MR elastomer in compression condition was analyzed by Liao *et al.* experimentally and theoretically. They found that, with the enhancement of external magnetic field and precompression force, the normal force of MR elastomer shows an increasing trend.^17^

In the field of engineering application of MR devices, the external loading condition that applied to MR device are mainly classified into three types, *i.e.* static load, quasi-static load and dynamic load, which are often differentiate by loading rate (shear rate). The shear rate of the dynamic load is often higher than 10^−1^ s^−1^, while the shear rate boundary of quasi-static load varies from 10^−4^ s^−1^ to 10^−1^ s^−1^.^23,24^ To date, the investigations into the normal force of MR materials are only carried out under limited loading conditions, *i.e.*, static (shear rate< 10^−4^ s^−1^) and dynamic (shear rate> 10^−1^/s) loadings.^16,17,21^ Researches addressing the normal force of MR grease under quasi-static shear (10^−4^ s^−1^ < shear rate < 10^−1^ s^−1^) were rarely reported. For real-life engineering applications of MR grease dampers to civil infrastructures, *i.e.*, building and bridge, MR grease is often subjected to the combination of large deformation and quasi-static shear loadings. To ensure the effective vibration isolation performance of such adaptive devices, the study on the normal force of MR grease under quasi-static shear is of great significance.

In this paper, the normal force of MR grease under different quasi-static shear modes, *i.e.* quasi-static monotonic shear and cyclic shear, with large deformation will be discussed. Firstly, MR grease with various weight fraction of CI particles were prepared. Then the influence of the shear rate and temperature on the magnetic-induced normal force of MR grease with different CI particles content under quasi-static monotonic shear were tested and analyzed. Finally, the shear strain dependence magnetic-induced normal force under quasi-static cyclic shear were investigated in detail.

## 2. Experimental testing

### 2.1 Sample preparation

MR grease used in this paper consist of carbonyl iron (CI) particles dispersed in a commercial lithium-based grease. CI particles with polycrystalline structure are purchased from BASF (Germany) Ltd. The type of the CI particles is CN, and its Fe content is as high as 99.5%. In addition, the average diameter of CI particles is 6 μm. The commercial lithium-based grease are obtained from the Shell (China) Ltd, whose properties are shown in Table 1. The process of the preparation of MR grease is as follows: first, a specified amount lithium-based grease with NLGI 0 was heated to 80 °C and stirred at 500 rpm for 10 minutes. Then CI particles with different weight fractions were mixed with the stirred grease at 800 rpm until fully mixed. MR grease with CI particles weight fraction of 30%, 50% and 70% were prepared separately, as shown in Table 2.

**Table 1:** Properties of the commercial lithium-based grease

Product type	Gadus S2V220
NIGL grade	0
Soap base	Lithium
Kinematic viscosity @ 40 °C, cSt	220
Kinematic viscosity @ 100 °C, cSt	19
Base oil	Mineral oil
ASTM worked penetration @ 25 °C 0.1 mm	355–385

**Table 2:** MR grease with different weight percentage of CI particles

Samples	CI particles (wt%)	Lithium-based grease (wt%)
MRG-30	30	70
MRG-50	50	50
MRG-70	70	30

**Fig. 1:**
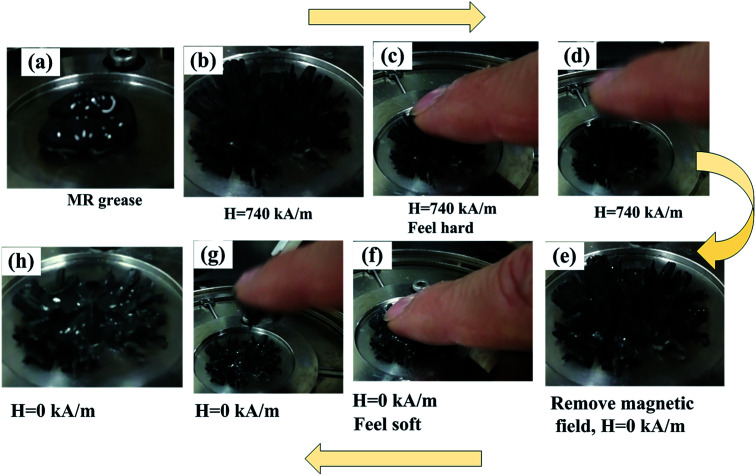
MR grease with and without magnetic field. MR grease (a) forms many sheet structures at 740 kA m^−1^ (b). Macroscopic behavior of the sheet structures resisting the external force at 740 kA m^−1^ (c) and (d). Macroscopic behavior of the sheet structures resisting the external force without magnetic field (e)–(h).

Fig. 1 shows the macroscopic behavior of MR grease with and without magnetic field. Fig. 1(a) is the photograph of the MR grease with CI particles weight fraction of 70%, which shows a semi-solid state in the zero-field condition. The behavior of MRG-70 with a magnetic field strength of 740 kA m^−1^ is shown in Fig. 1(b), it is found that many sheet structures are formed along the direction of magnetic field. In figure (c) and (d), when we touch these sheet-like structures with our hands, we feel very hard and the structures are not broken. Fig. 1(e) shows that the sheet-like structures can be maintained after removing the magnetic field, which is very different from the disappearance of the MR fluid microstructures.^25,26^. In figure (f), (g) and (h), in the absence of the magnetic field, when we touched the sheet structures again with our hands, we found that the retained structures were destroyed instantly, which indicates that the retained structures of MR grease are very fragile.

### 2.2 Experimental setup

**Fig. 2:**
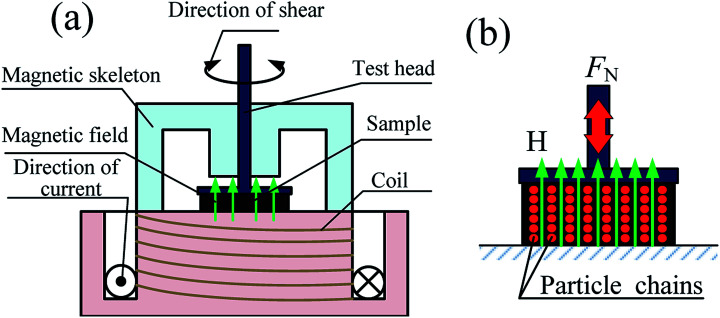
(a) Main configuration of the MCR 302 parallel-plate rheometer. (b) Schematic diagram of the magneto-induced normal force in the testing system.

The magnetic properties of CI particles used in this paper is measured by using the Vibrating Sample Magnetometer (VSM, Lakeshore, 7404 Series) at room temperature. The normal force of MR grease under quasi-static shear was tested using a parallel-plate rheometer (type MCR 302, Anton Paar Co., Austria) equipped with magnetorheological module (Type MRD180, Anton Paar Co., Austria) and temperature-control accessory (Type F25, Julabo Technology Co, Germany). Fig. 2(a) shows the main configuration of MCR 302 rheometer, the diameter of the parallel-plate under the test head is 20 mm. A 0.318 ml sample of MR grease was placed on the base plate and the gap between the plate was set as 1 mm. The magnitude of the magnetic field strength passing through the gab can be controlled by the coil current. Fig. 2(b) shows the schematic diagram of the magneto-induced normal force in the testing system. CI particles distributed in MR grease are arranged in the direction of external magnetic field to form a chain structure, which make the test head subjected to a upward force (normal force). The maximum normal force measurement range of MCR 302 rheometer is 50 N.

**Fig. 3:**
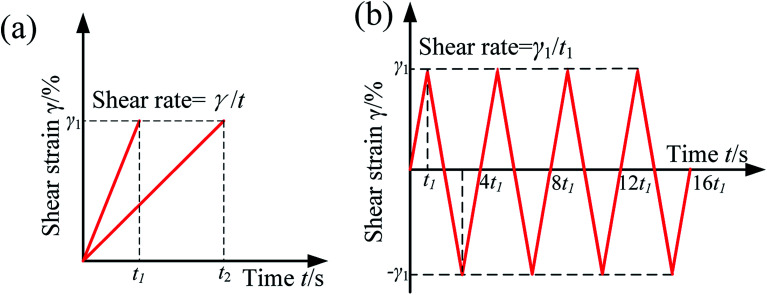
Test principle of the quasi-static monotonic shear (a) and quasi-static cyclic shear (b).

In this paper, there are two modes, *i.e.* monotonic and cyclic shear, were used to test the normal force of MR grease with different CI particles content under various magnetic field. Fig. 3(a) shows the test principle of the quasi-static monotonic shear. In this mode, the shear strain that applied to the sample through the rheometer using displacement control was linearly increase from initial 0% to maximun 100% in different time such as 10 s, 20 s, 30 s, 50 s and 200 s. The corresponding quasi-static shear rate can be obtained by dividing the maximum shear strain by the time, which is 0.1 s^−1^, 0.05 s^−1^, 0.033 s^−1^, 0.02 s^−1^ and 0.005 s^−1^ respectively. For the quasi-static monotonic shear of MRG-70 under different magnetic field strength, four different magnetic field, *i.e.* 0 kA m^−1^, 96 kA m^−1^, 194 kA m^−1^ and 391 kA m^−1^, were used and tested at a fixed shear strain, 100%, and shear rate, 0.05 s^−1^. For the quasi-static monotonic shear of MR grease with different CI particles content, the sample used in the test include MRG-30, MRG-50 and MRG-70. The test conduct at fixed shear strain, 100% and shear rate, 0.05 s^−1^. For the quasi-static monotonic shear of MRG-70 under different shear rate, the test was carried out at four different shear rate, *i.e.*, 0.1 s^−1^, 0.033 s^−1^, 0.02 s^−1^ and 0.005 s^−1^, and the shear strain were fixed at 100%. For the quasi-static monotonic shear of MRG-70 under different temperature, the temperature was conducted at four different types, *i.e.*, 5 °C, 25 °C, 45 °C and 65 °C. The shear strain and shear rate were set as 100% and 0.05%. Each set of above tests was carried out at 0 kA m^−1^, 96 kA m^−1^, 194 kA m^−1^ and 391 kA m^−1^, respectively.

Fig. 3(b) shows the test principle of the quasi-static cyclic shear. In this mode, shear strain applied to the sample changes in the form of a triangular wave. For the quasi-static cyclic shear test of MRG-70 under different magnetic field, two types of magnetic field strength, *i.e.*, 0 kA m^−1^ and 96 kA m^−1^, were used and tested at a shear strain, 100%, and shear rate, 0.05 s^−1^. For the quasi-static cyclic shear test of MRG-70 under different shear strain, five different shear strain, *i.e.* 20%, 40%, 60%, 80% and 100%, were applied in different time allows the shear rate to be fixed at 0.05 s^−1^. The magnetic field strength was kept as 96 kA m^−1^. In all tests, the sample was pretreatment in a shear flow and stand for 30 s. All the experiment are performed at 25 °C unless otherwise stated. Moreover, all the tests were conducted five times to ensure the reproducible of the date.

## 3. Result and discussion

### 3.1 Magnetic properties of the CI particles

**Fig. 4:**
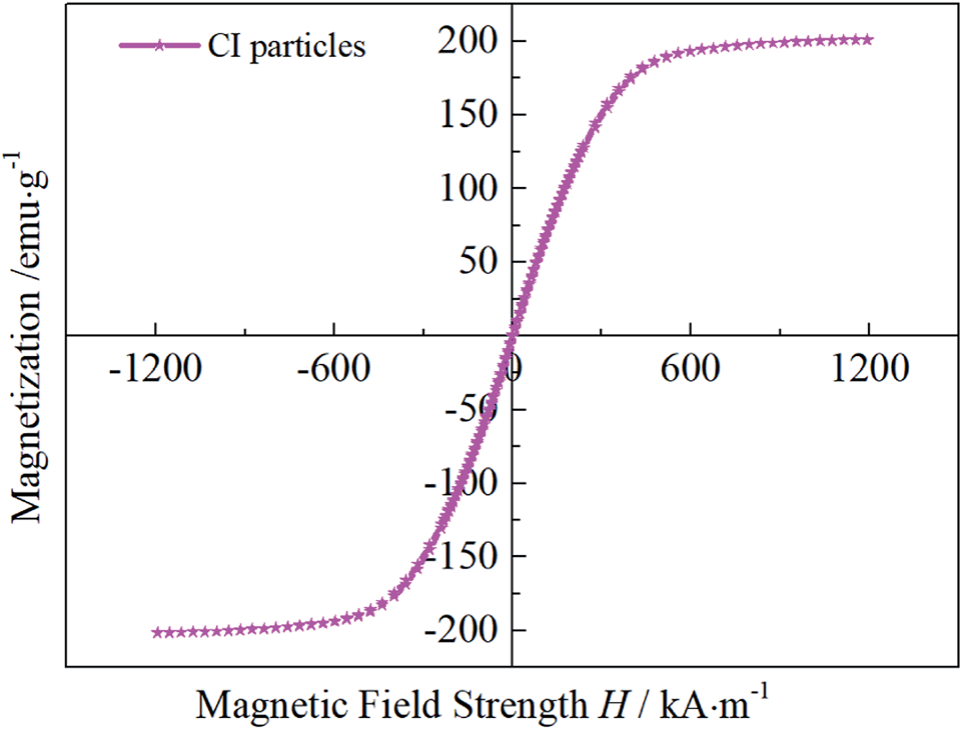
Magnetization curve of the CI particles.

The magnetization curve of the CI particles used in this paper is shown in Fig. 4. In the initial stage, the magnetization of the CI particles shows a linear increase with the increase of magnetic field strength. When the external magnetic field is higher than 400 kA m^−1^, the magnetization curve tends to saturate. The highest magnetization of CI particles used in this paper display is around 206 emu g^−1^.

### 3.2 Normal force of MR grease under quasi-static monotonic shear

#### 3.2.1 Quasi-static monotonic shear for MRG-70 under different magnetic field

**Fig. 5:**
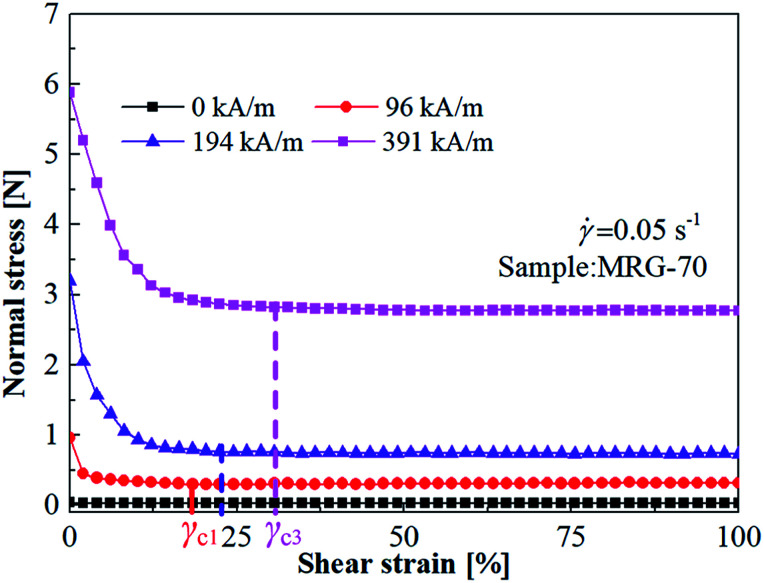
The shear strain dependence normal force of MRG-70 under different magnetic filed.

Fig. 5 shows the normal force of MRG-70 as a function of shear strain at the shear rate of 0.05 s^−1^ under different magnetic filed. When an external magnetic field was not applied, normal force of MRG-70 remains constant along with the shear strain. In the presence of magnetic field strength, there is a critical strain amplitude, *γ*_c_, that divide the relationship between the normal force and shear strain into two stages. When the shear strain is smaller than *γ*_c_, normal force presents a rapid decrease trend. However, normal force remains almost constant at the shear strain higher than *γ*_c_. This phenomenon is consistent with the research of See *et al.* on MR fluid.^19^ A possible reason for the phenomenon can be attributed to the microstructure change of MR grease under the shear and magnetic field. Under the external magnetic field strength, CI particles distributed in MR grease aggregate and form chains or clusters structure along the direction of magnetic field strength, when the shear strain increase from 0 to *γ*_c_, the chains or clusters structure gradually rupture, causing the normal force to decrease. As the shear strain increases above *γ*_c_, the rupturing and rebuilding of the chains or clusters microstructure reach a dynamic equilibrium under the action of the external shear and magnetic field, the microscoptic performance is that normal stress remains constant.

#### 3.2.2 Quasi-static monotonic shear for MR grease with different CI particles content

**Fig. 6:**
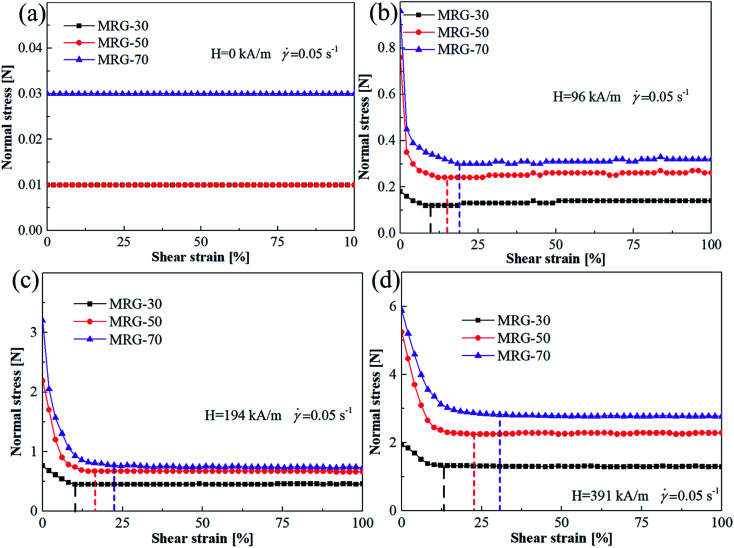
Normal force as a function of shear strain for different MR grease at shear rate of 0.05 s^−1^ (a) H = 0 kA m^−1^; (b) H = 96 kA m^−1^; (c) H = 194 kA m^−1^; (d) H = 391 kA m^−1^.

Fig. 6 shows the normal force *versus* shear strain curves for three types of MR grease, *i.e.*. MRG-30, MRG-50 and MRG-70, under different magnetic field strength. In the absence of the magnetic field, the normal force of MR grease with different CI particles are all very small and constant. From Fig. 6(b), (c) and (d), when the magnetic field strength was applied, the changing regularity of the normal force of MRG-30 and MRG-50 with the shear strain are similar to that of MRG-70, *i.e.*, with the increasing of the shear strain, normal force decreases first and then tends to constant. The critical strain amplitude for three types of MR grease under different magnetic field shown in Fig. 6 are presented in Table 3. From Table 3, when the magnetic field strength increase from 96 kA m^−1^ to 391 kA m^−1^, the magneto-induced increase of critical strain amplitude of MRG-30, MRG-50 and MRG-70 are 4.8%, 8.2% and 12.1%, respectively. In addition, the particles content-induced increase of the critical strain amplitude of MR grease presented an increasing trend with the enlargement of the external magnetic field, *i.e.* 10.3%, 12% and 17.6% for 96 kA m^−1^, 194 kA m^−1^ and 391 kA m^−1^. The above phenomenon indicate that the critical strain amplitude of MR grease increase with the increase of the CI particles content and magnetic field strength, which is attributed to the stronger chains or cluster structures are formed at higher magnetic filed and CI particles content.

**Table 3:** The critical strain amplitude for three types of MR grease under different magnetic field

Sample	Critical strain amplitude *γ*_c_			Magneto-induced increase *Δ = γ*_391 kA m^−1^_*− γ*_96 kA m^−1^_
	Magnetic field strength H			
	96 kA m^−1^	194 kA m^−1^	391 kA m^−1^	
MRG-30	8.2%	10.2%	13%	4.8%
MRG-50	14.3%	16.3%	22.5%	8.2%
MRG-70	18.5%	22.2%	30.6%	12.1%
Particles content-induced increase *Δ = γ*_MRG-70_*− γ*_MRG-30_	10.3%	12%	17.6%	

#### 3.2.3 Quasi-static monotonic shear for MRG-70 under different shear rate

**Fig. 7:**
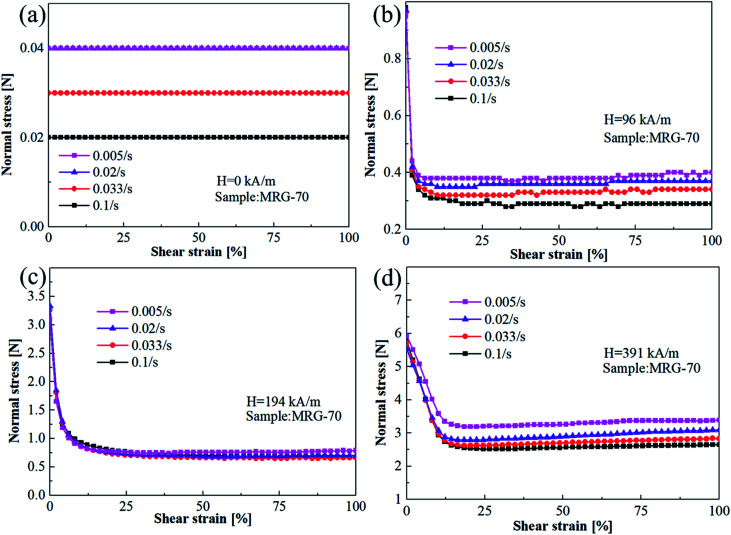
The shear rate dependence of normal force for MRG-70 under different magnetic field strength (a) H = 0 kA m^−1^; (b) H = 96 kA m^−1^; (c) H = 194 kA m^−1^; (d) H = 391 kA m^−1^.

To evaluate the shear rate dependence of normal force *versus* strain curves under different magnetic field strength, the normal force of MRG-70 as a function of shear strain under different shear rate and magnetic field strength are presented in Fig. 7. It is observed from Fig. 7 that, under a given shear strain, normal force exhibits a decreasing trend with the increase of shear rate, and this change regularity is affected by the external magnetic field. The above phenomenon is opposite to the relationship that shear stress of MR plastomer increases with the shear rate.^27^ Moreover, from Fig. 7(a), in the absence of magnetic field strength, the normal force of MRG-70 under different shear rate keeps constant along with the shear strain. From Fig. 7(b), (c) and (d), under the application of the magnetic field strength, the normal force of MRG-70 under different shear rate are all demonstrate a decrease at the beginning stage of the shear strain, and as the magnetic field increases, the rate of decrease becomes slower. Another interesting phenomenon from Fig. 7 is that, under the small magnetic field strength such as 96 kA m^−1^, the critical strain amplitude of normal force–strain curves, *γ*_c,_ is increasing with the shear rate, and this increasing trend is not obvious with the increase of the magnetic field.

#### 3.2.4 Quasi-static monotonic shear for MRG-70 under different temperature

**Fig. 8:**
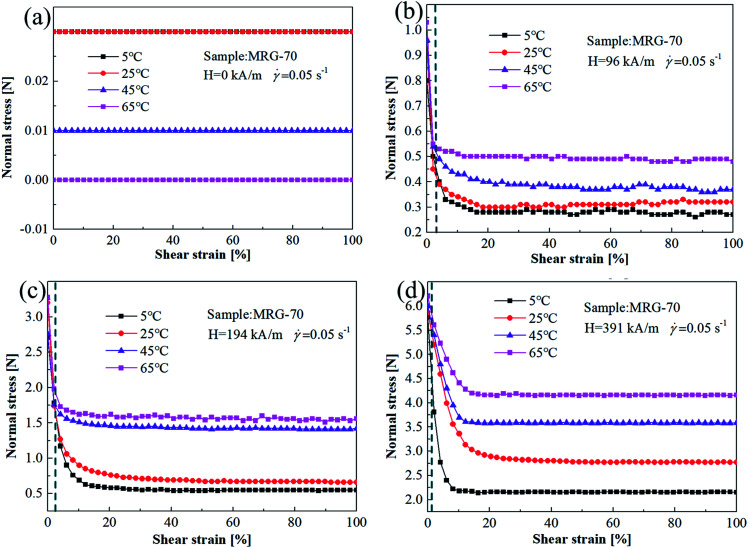
The normal force *vs.* shear strain curves for MRG-70 under different temperature (a) H = 0 kA m^−1^; (b) H = 96 kA m^−1^; (c) H = 194 kA m^−1^; (d) H = 391 kA m^−1^.

Fig. 8 shows the change of normal force of MRG-70 with shear strain under different temperature and magnetic field strength. As the increase of the shear strain, normal force of MRG-70 under different temperature remains constant in the absence of magnetic field, but decreases first and then tends to unchanged at the applied magnetic field. On the other hand, it also can be seen from Fig. 8(b), (c) and (d) that, when the shear strain is smaller than a critical value, temperature has no effect on normal force and the corresponding critical value decrease with the increase of magnetic field, *i.e.* 3.2%, 2.4% and 0.9% for 96 kA m^−1^, 194 kA m^−1^ and 391 kA m^−1^. With the increase of strain amplitude, the normal force of MRG-70 demonstrates an increasing tendency along with the temperature, and this increasing tendency is correlated with the magnetic field strength. The reason for this is that, the solid-like grease matrix has an effect on the chain formation of CI particles, as the temperature increase, the viscosity of the grease matrix decreases which benefits the chain formation of CI particles distributed in the matrix. Meanwhile, the magnetic force between the CI particles also enhanced by the increase of magnetic field strength. Therefore, the normal force of MR grease can be enhanced at high temperature and magnetic field strength.

### 3.3 Normal force of MR grease under quasi-static cyclic shear

#### 3.3.1 Quasi-static cyclic shear for MRG-70 under different magnetic field

**Fig. 9:**
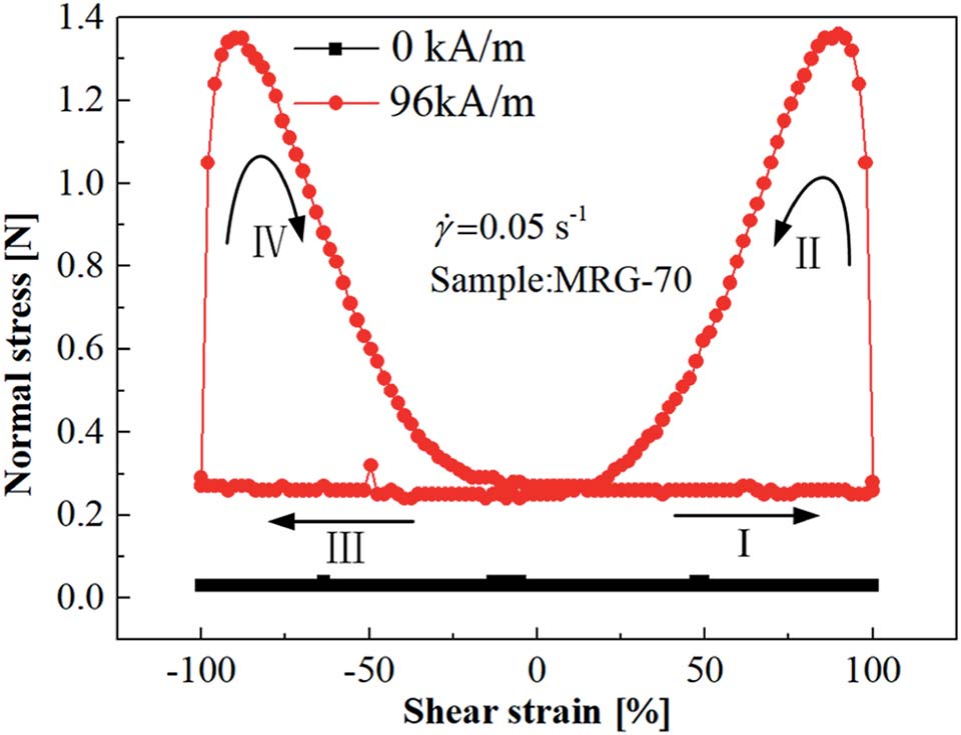
The cyclic normal force–strain curves of MRG-70 under different magnetic field strength: I, loading stage; II, unloading stage; III, reverse loading stage; IV, reverse unloading stage.

To investigated the normal force behavior of MR grease under quasi-static cyclic shear, the shear strain linearly increased from 0 to 100%(I: loading stage) and then decreased to 0(II: unloading stage). After that, shear strain reversely loaded to −100% (III: reverse loading stage) and then restored to 0 (IV: reverse loading stage). The time that used for every stage is 20 s, and the corresponding quasi-static shear rates is 0.05 s^−1^. To ensure the stability of the material properties in each case, at least 5 cycles were measured for each cycle loading. Fig. 9 shows the normal force *versus* shear strain curves of MRG-70 at the quasi static shear rate of 0.05 s^−1^ under different magnetic field strength. We only discuss the loading stage I and unloading stage II of the cyclic curve here due to the symmetry of the cyclic normal force *versus* shear strain curve. When the external magnetic field was not applied, the normal force of MRG-70 in stage I, II, III and IV are the same and tend to 0. Under the magnetic field strength of 96 kA m^−1^, the normal force at different stages presents a big difference. At the loading stage I, the normal force of MRG-70 nearly maintains constant with the increase of shear strain, but in the unloading stage II, the normal force of MRG-70 exhibit peak phenomenon at the beginning of unloading stage. This is because of that, under the action of magnetic field and shear, the chain structures reconstruct and rupture of MR grease in loading stage I has reached an equilibrium state after multiple shear loading cycles. At the beginning of shear strain decrease from 100% to 0, the internal broken chain structures are gradually reconstructing, which is beneficial to the increase of normal force. As the further decrease of shear strain, the continuous reconstruction of the particle chain is squeezed by the parallel-plate, which in turn causes the chain structure to rupture. The coupled influence leads to the peak phenomenon of normal force for MR grease in unloading stage II. In addition, the normal force behavior of MR grease in reverse loading stages III and reverse unloading stage IV is similar to loading stage I and unloading stage II, respectively.

#### 3.3.2 Quasi-static cyclic shear for MRG-70 under different shear strain

**Fig. 10:**
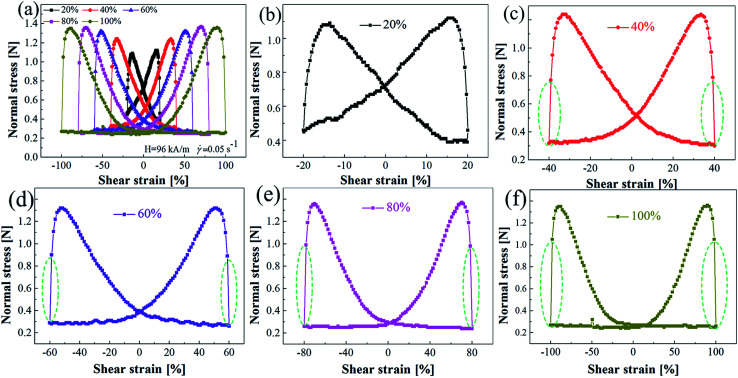
(a) Cyclic normal force–strain curves for MRG-70 under five different shear strain. (b)–(f) is a single representation of five types of the cyclic normal force–strain curves shown in (a), respectively.

Fig. 10 shows the cyclic normal force *versus* strain curves for MRG-70 under five different shear strain. It can be seen from Fig. 10(a) that, as the applied shear strain increased, the peak normal force of the cyclic curves demonstrates an increase trend, with the applied shear strain higher than 80%, the peak normal force of the cyclic curves nearly maintains constant, *i.e.* 1.12 N, 1.24 N, 1.32 N, 1.36 N and 1.36 N for 20%, 40%, 60%, 80% and 100%, respectively. From Fig. 10(b)–(d), at the applied shear strain of 20%, the cyclic normal force *versus* strain curve displays a butterfly-like shape, as the increase of the applied shear strain, the intersection point of the cyclic normal force *versus* strain curve gradually shifts downward, causing the butterfly-like shape of cyclic curve to disappear. In addition, the area enclosed by the cyclic normal force *versus* strain curves increases with the increase of applied strain, which can be attributed to the phase difference between the applied shear strain and structural strain.^21^

Another interesting phenomenon we can find from the cyclic normal force–strain curves marked by the green dotted line in Fig. 10(b)–(f) is that, when the shear strain is higher than 40%, normal force exhibits an abrupt change during the transition from loading stage I to unloading stage II (or reverse loading stages III to reverse unloading stage IV), and the abrupt change rate increase along with the increase of shear strain, *i.e.* 163%, 242%, 303% and 308% for 40% 60%, 80% and 100%, respectively. This has never been reported in MR fluid or MR polymer. The possible reason may be induced by the special viscoplastic properties of grease matrix of MR grease.

## 4. Conclusion

In this paper, three types of MR grease were prepared by dispersing different weight fraction of CI particles into lithium-based grease. The normal force *versus* strain curves of these three MR samples under different test conditions, *i.e.* shear rate, temperature and magnetic field strength, were obtained by using quasi-static monotonic and cyclic shear test. In the quasi-static monotonic shear test, the results demonstrate that, a critical strain amplitude, *γ*_c_, divides the relationship between the normal force and shear strain into two stages. When the shear strain is smaller than *γ*_c_, normal force presents a rapid decrease trend with the increase of shear strain. However, normal force remains almost constant at the shear strain higher than *γ*_c_, and the critical strain is affected by the magnetic field strength, CI particles content and shear rate. Moreover, when the shear strain is smaller than a critical value, temperature has no effect on normal force. With the increase of strain amplitude, the normal force of MRG-70 demonstrates an increasing tendency along with the temperature, and this increasing tendency is correlated with the magnetic field strength. For the quasi-static cyclic shear test, the results indicate that, at the unloading stage II, the normal force of MRG-70 exhibit peak phenomenon at the beginning of the unloading stage. As the applied shear strain increased, the peak normal force of the cyclic curves demonstrates an increasing trend, with the applied shear strain higher than 80%, the peak normal force of the cyclic curves nearly maintains constant. In addition, when the shear strain is higher than 40%, normal force exhibits an abrupt change during the transition from loading stage I to unloading stage II(or reverse loading stages III to reverse unloading stage IV).

## Conflicts of interest

There are no conflict to declare.

## Supplementary Material
